# Oncocytic lipoadenoma of the parotid gland: a case report and a review of the literature

**DOI:** 10.1093/jscr/rjae533

**Published:** 2024-11-22

**Authors:** Jood K Alotaibi, Turki Mohammed Almuhaimid, Ghada Abdallah Moumneh

**Affiliations:** College of Medicine, Imam Abdulrahman Bin Faisal University, Dammam, Eastern Province 32210, Saudi Arabia; Department of Otolaryngology-Head and Neck, King Fahad Specialist Hospital, Dammam, Saudi Arabia; Department of Pathology, Dr Sulaiman Alhabib Hospital, Alkhobar, Saudi Arabia

**Keywords:** oncocytic, parotid, lipoadenoma

## Abstract

Oncocytic lipoadenomas are a rare sub-category of fat-containing tumors of the salivary glands. These tumors are characterized by their oncocyte-predominant epithelial component admixed with mature adipocytes. This condition has been rarely reported to affect the parotid and the submandibular glands. We report a case of a 69-year-old male who presented to our otolaryngology outpatient department with a complaint of a gradually growing right-sided infra-auricular neck mass. A surgical full mass excision was performed, and a histopathological evaluation yielded a tumor with oncocytes organized in tubular structures. This paper presents the fourth unusual case of a giant oncocytic lipoadenoma of the parotid gland measuring 11.5 × 10.5 × 11.5 cm in anteroposterior, transverse, and cranial planes, respectively.

## Introduction

Parotid gland oncocytic lipoadenoma is a rare mixed lipoepithelial tumor characterized by oncocyte-predominant epithelial components intermingled with mature adipocytes [1]. The exact etiology remains unclear, and due to its rarity, it was not included in the 2005 World Health Organization (WHO) classification of salivary gland tumors [2]. This condition has a male predominance, primarily affecting older individuals, and the majority were complaining of an infra-auricular mass measuring less than 5 cm [2]. A definitive histopathological evaluation is needed to establish the diagnosis. In this paper, we present a case of a sixty-nine-year-old male who presented with a right infra-auricular giant mass measuring 11.5 × 10.5 × 10.5, which was later confirmed to be parotid gland oncocytic lipoadenoma.

## Case presentation

**Figure 1:**
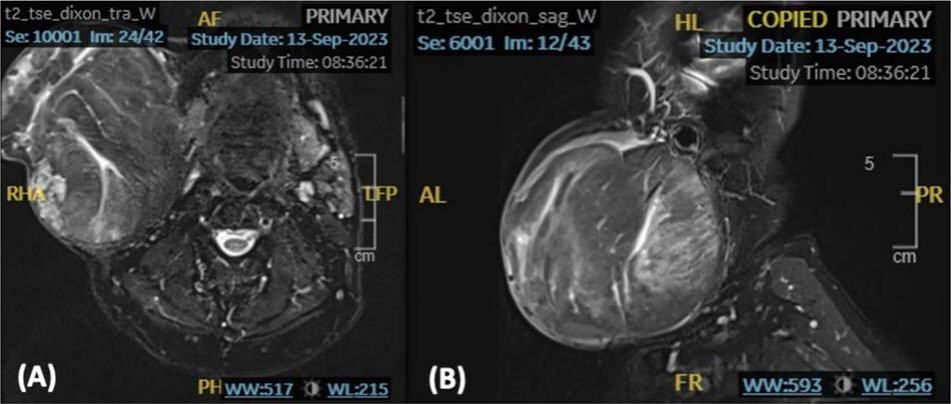
An axial (A) and sagittal (B) T2-weighted MRI showing a rounded well-defined heterogeneous lesion with soft tissue signal.

We present a case of a medically free sixty-nine-year-old male patient who presented to the otolaryngology outpatient department with a complaint of right-sided infra-auricular neck mass. The patient claims that the neck mass has been present for more than ten years; however, it has increased in size over the past four months prompting him to seek medical attention. Upon further history taking, the mass was slowly progressive, painless, and not associated with trismus, facial asymmetry, signs of inflammation, or other neck mass. On examination, the patient was vitally stable and looking well, with a nontender right-sided neck mass extending from the tail of the parotid superiorly up to the level of the cricoid inferiorly. The mass was well-defined, mobile, and multilobulated, approximately measuring 10 × 10 cm in size. A T2-Weighted Fast Spin Echo (FSE) noncontrast magnetic resonance imaging (MRI) of the neck demonstrated a large discrete rounded heterogeneous lesion, with soft tissue signal, noted at the right parotid region measuring 11.5 × 10.5 × 11.5 cm in anteroposterior, transverse, and cranial planes, respectively (Fig. 1). A significant mass effect on adjacent structures causing elevation and stretching of the overlying neck skin, sternocleidomastoid, submandibular gland, and masseter muscle was noted. However, the right mandible appears preserved with no lymph node involvement.

**Figure 2:**
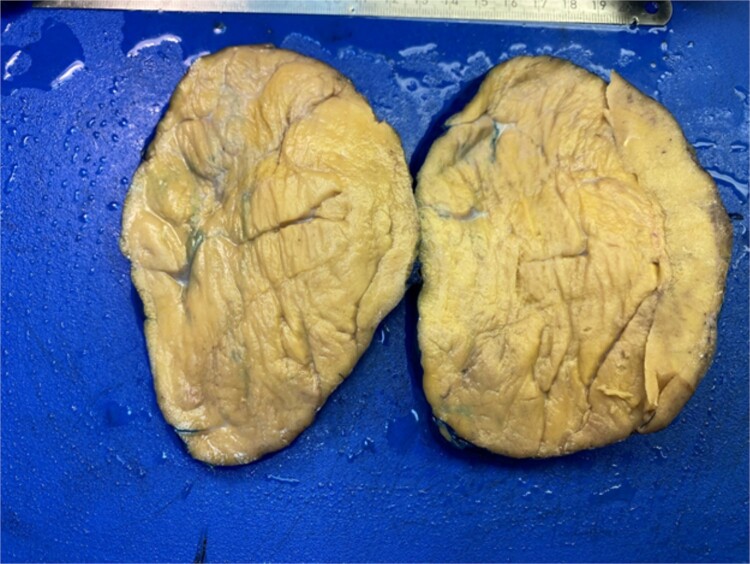
Gross pathology of the tumor.

**Figure 3:**
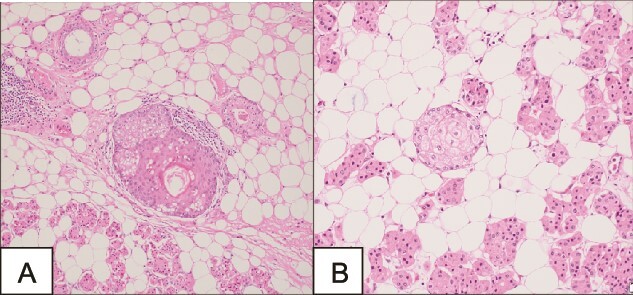
Microscopy demonstrating a tumor of prominent oncocytes organized in tubular structures, admixed with fatty tissue containing mature adipocytes.

A surgical full mass excision was performed (Fig. 2). A histopathological evaluation of the lesion revealed a tumor of prominent oncocytes organized in tubular structures, admixed with fatty tissue containing mature adipocytes in varying proportions. The tumor was encapsulated by a slightly thick, dense fibrous covering. Notably, the oncocytic tubules were mainly localized at the periphery of the tumor. Dilated ducts showed variable degrees of squamous metaplasia with keratinization and less prominent sebaceous metaplasia (Fig. 3). The patient had an uneventful postoperative course and a follow-up appointment one week later. During the visit, the patient was in good health and the facial nerve was intact.

## Discussion

The histopathological spectrum of fat-containing tumors of the salivary glands varies from pure lipomas originating from epithelial and myoepithelial origins to mixed lipoepithelial lesions [1]. A subdivision of the mixed lipoepithelial tumors is known as oncocytic lipoadenoma, first reported by Hirokawa *et al.* in 1998 [2]. Due to its scarcity, this entity was not included in the 2005 WHO classification system of salivary gland tumors [2]. Microscopically, these tumors show a predominance of oncocytic-like epithelial components with a variable degree of fat cells [1]. The pattern of the oncocytes is either intermingled with the adipocytes or forms a nodular oncocytic discrete lesion in a lipomatous background [1]. This uncommonly reported condition is clinically defined as a benign salivary gland tumor that develops gradually and progressively in its course with a predilection to the parotid gland, and less frequent occurrence in the submandibular gland [1]. In our case, the oncocytic lipoadenoma was originating from the tail of the parotid gland.

According to the literature, we have identified twenty-six cases of parotid gland oncocytic lipoadenoma, the first reported occurrence of an oncocytic lipoadenoma in the parotid gland was reported by Kato *et al.* in 2000 (Table 1). The age of these cases ranged from seven to eighty-nine years. The literature provided adequate evidence that most cases were complaining of a neck mass measuring less than 5 cm, where the smallest size was reported to be 1.9 cm and the largest was measuring 15 cm. In our case, we are reporting the fourth unusual giant oncocytic lipoadenoma measuring 11.5 cm. The signs and symptoms of parotid gland oncocytic lipoadenoma are associated with the local enlargement of this condition, where patients clinically present with a slowly growing infra-auricular mass, not associated with local skin changes or lymphadenopathy.

**Table 1:** Summarized cases of parotid gland oncocytic lipoadenoma.

Reference	Age	Gender	Site	Size (in cm)
Klieb HB [1]	47 years old	Female	Left parotid gland	3
Shakya D [2]	46 years old	Male	Right parotid gland	15
Chahwala Q [3]	50 years old	Female	Left parotid gland	14
Devadoss CW [4]	50 years old	Female	Left parotid gland	13.5
Kato M [5]	57 years old	Female	Right parotid gland	4.5
Lau SK [6]	61 years old	Male	Left parotid gland	2
	83 years old	Male	Right parotid gland	2.5
	67 years old	Male	Right parotid gland	4
	40 years old	Female	Right parotid gland	4
	56 years old	Male	Left parotid gland	3.5
	65 years old	Male	Left parotid gland	1.9
	65 years old	Male	Right parotid gland	3.5
Sureja VP [7]	59 years old	Male	Right parotid gland	5.5
Chi CL [8]	71 years old	Male	Right parotid gland	4.2
Mitsimponas KT [9]	55 years old	Female	Left parotid gland	2.7
Ashraf MJ [10]	56 years old	Female	Right parotid gland	3
Agaimy A [11]	63 years old	Male	Parotid gland	4.5
	29 years old	Male	Parotid gland	4.5
	54 years old	Female	Parotid gland	2.9
	7 years old	Female	Parotid gland	Not available
	89 years old	Female	Parotid gland	4.2
	55 years old	Male	Parotid gland	2.7
Aouad R [12]	38 years old	Male	Left parotid gland	3.8
Illie M [13]	64 years old	Male	Left parotid gland	5
McNeil ML [14]	73 years old	Male	Parotid gland	Not available
Tokyol C [15]	56 years old	Male	Left parotid gland	4

This condition has a wide variety of differential diagnoses including sialolipomas, pleomorphic adenomas, sclerosing polycystic adenosis, and oncocytic metaplasia [2]. Thus, a high clinical suspicion after taking a thorough history and physical examination needs to be augmented by radiological and histopathological evaluation. A CT scan is performed to show a heterogeneous lesion with a fat component narrowing the list of differentials into lipomas and lipoadenomas [1]. An MRI can also be performed, showing an intermediate-signal intensity heterogeneous lesion in both T1 and T2 [9]. However, these radiological signs are nonspecific; thus, a histopathological evaluation is needed to reach a definitive diagnosis. Microscopically, lipoadenomatous tumors are encapsulated with micronodules or islands of dark and light oncocytes, with a component of residual normal acini [9]. In some cases, a sebaceous metaplasia could also be detected [5]. All the reported cases were surgically managed with lateral parotidectomy or local excision of the tumor [9].

## Conclusion

Given the rarity of this condition, it could be easily misdiagnosed. Therefore, in this paper, we are reporting an unusual case of a giant oncocytic lipoadenoma, aiming to contribute to the existing literature.

## References

[1] Klieb HB , Perez-Ordoñez B. Oncocytic lipoadenoma of the parotid gland with sebaceous differentiation. Study of its keratin profile. Virchows Arch 2006;449:722–5. 10.1007/s00428-006-0317-z. 17091251

[2] Shakya D , Nepal A. An extremely rare case of giant oncocytic adenolipoma of the parotid gland. Clinical Case Reports 2020;8:2390–4. 10.1002/ccr3.3151. 33363747 PMC7752572

[3] Chahwala Q , Siddaraju N, Singh N, et al. Fine needle aspiration cytology of oncocytic lipoadenoma of the parotid gland: report of a rare case. Acta Cytol 2009;53:437–40. 10.1159/000325348. 19697732

[4] Devadoss CW , Murugan P, Basu D, et al. Oncocytic lipoadenoma of the parotid gland: report of a rare case. J Clin Diagn Res 2012;6:1076–8.

[5] Kato M , Horie Y. Oncocytic lipoadenoma of the parotid gland. Histopathol 2000;36:285–6. 10.1046/j.1365-2559.2000.0872e.x. 10809601

[6] Lau SK , Thompson LD. Oncocytic lipoadenoma of the salivary gland: a clinicopathologic analysis of 7 cases and review of the literature. Head Neck Pathol 2015;9:39–46. 10.1007/s12105-014-0543-7. 24737102 PMC4382475

[7] Sureja VP , Tagore KR. Oncocytic sialolipoma of parotid gland: case report and literature review. Indian J Pathol and Microbiol 2023;66:591–3. 10.4103/ijpm.ijpm_323_21. 37530346

[8] Chi CL , Kuo TT, Lee LY. Oncocytic lipoadenoma: a rare case of parotid gland tumor and review of the literature. J Pathol and Translational Med 2015;49:144–7. 10.4132/jptm.2014.02.10. PMC4367110 25812735

[9] Mitsimponas KT , Agaimy A, Schlittenbauer T, et al. Oncocytic lipoadenoma of the parotid gland: a report of a new case and review of the literature. Int J Clin Exp Pathol 2012;5:1000–6. 23119120 PMC3484499

[10] Ashraf MJ , Azarpira N, Anbardar MH, et al. Oncocytic lipoadenoma of the parotid gland: cytological findings and differential diagnosis on fine-needle aspiration. Diagn Cytopathol 2015;43:72–4. 10.1002/dc.23135. 24591268

[11] Agaimy A , Ihrler S, Märkl B, et al. Lipomatous salivary gland tumors: a series of 31 cases spanning their morphologic spectrum with emphasis on sialolipoma and oncocytic lipoadenoma. Am J Surg Pathol 2013;37:128–37. 10.1097/PAS.0b013e31826731e0. 23232852

[12] Aouad R , Matar N, Sader-Ghorra C, et al. Pathology quiz case 1. Archives of Otolaryngology–Head & Neck Surgery 2008;134:446. 10.1001/archotol.134.4.446. 18427016

[13] Ilie M , Hofman V, Pedeutour F, et al. Oncocytic lipoadenoma of the parotid gland: immunohistochemical and cytogenetic analysis. Pathology-Research and Practice 2010;206:66–72. 10.1016/j.prp.2009.02.008. 19346081

[14] McNeil ML , Bullock MJ, Trites JR, et al. Oncocytic lipoadenoma of the parotid gland with sebaceous differentiation in a 73-year-old male. Journal of Otolaryngology-Head & Neck Surgery= Le Journal d'oto-rhino-laryngologie et de chirurgie cervico-faciale 2010;39:E48–50. 20828502

[15] Tokyol Ç , Dilek FH, Aktepe F, et al. Oncocytic lipoadenoma of the parotid gland: a case report with fine needle aspiration cytology findings. Kulak Burun Bogaz Ihtisas Dergisi: KBB= Journal of Ear, Nose, and Throat 2010;20:146–9. 20465541

